# Optimal placement criteria of hybrid mounting system for chassis in future mobility based on beam-type continuous smart structures

**DOI:** 10.1038/s41598-023-29379-1

**Published:** 2023-02-09

**Authors:** Yang Qiu, Dongwoo Hong, Byeongil Kim

**Affiliations:** School of Mechanical Engineering, Yeungnam University, Gyeongsan, 38541 Republic of Korea

**Keywords:** Engineering, Mechanical engineering

## Abstract

Recently, research into the development of hybrid and electric vehicles has been vigorously undertaken, indicating a trend toward the replacement of internal combustion engine vehicles. However, while high efficiency and light weight are crucial in the development of vehicles, they increase the excitation force of the engine. In addition, sensor placement in future mobility is very important since it causes malfunctioning of autonomous driving systems when the location and orientation of sensors are changed due to excessive vehicle vibration. To reduce the structure-borne noise and vibration caused by engine excitation, an active engine mounting system must be installed in an optimal location. Thus, in this study, to determine the optimal location for an active engine mounting system applied to a beam structure, a series of simulations with two different methodologies are performed. The overall beam structure with two active mounting systems is modeled based on the lumped parameter model. To determine the optimal position of the active mounting system, it is moved to equal intervals, and the force and phase of the active mounts at each location combination are calculated based on static and dynamic methods. The optimal position is suggested such that the vibration reduction is maximized, while the applied force is minimized. Additionally, a feasibility experiment is conducted to validate the proposed criteria and confirm the simulation results. The results demonstrate that the optimal location of the active engine mounting system with a minimized force requirement and maximized vibration reduction can be identified.

## Introduction

**Figure 1:**
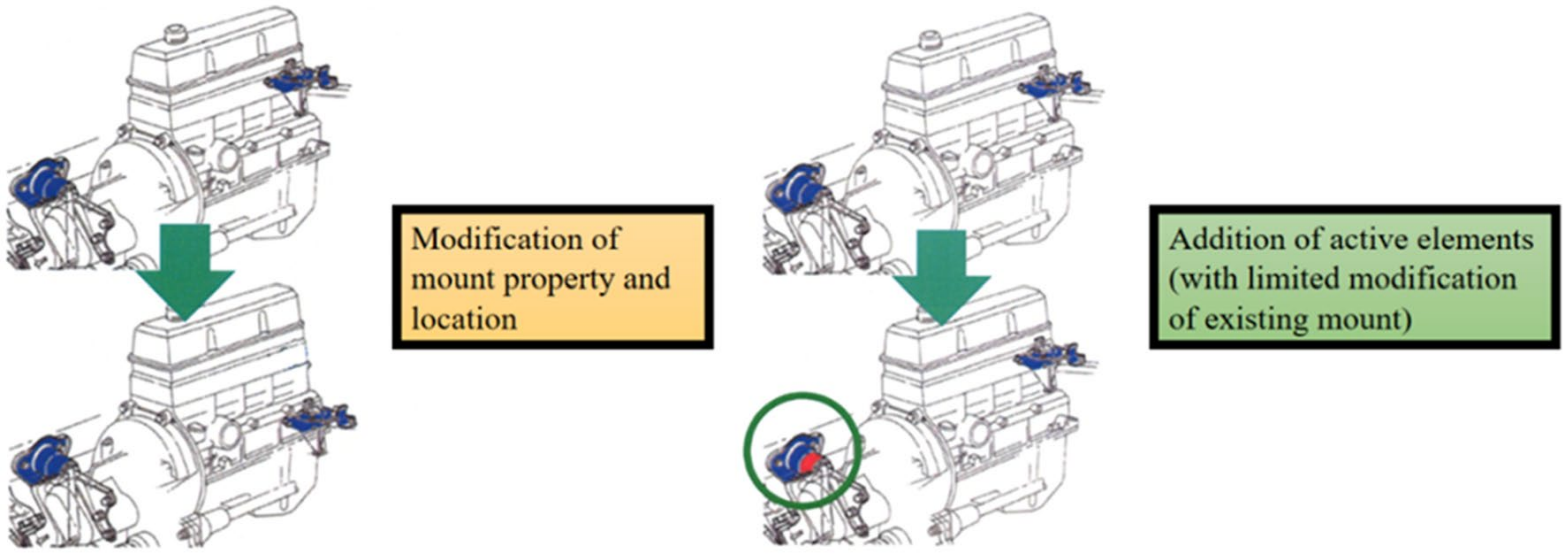
Two different ways of improving NVH performance of powertrain mounting system.

Recently, hybrid and electric vehicles are being actively researched and developed in order to replace conventional internal combustion engine vehicles. Compared with traditional cars, this car has higher requirements for power and weight, and at the same time increases the excitation force of the engine in the mid-frequency range. In addition, sensor placement in future mobility is very important since it causes malfunctioning of autonomous driving systems when the location and orientation of sensors are changed due to excessive vehicle vibration. The engine excitation force is impacted by other components, such as the power transfer unit, sub-frame, and body, through the engine mounts. Furthermore, it produces structure-borne noise that may cause considerable discomfort to the driver. The current method of reducing the noise, vibration, and harshness (NVH) under various operating conditions has limitations. Thus, to overcome these limitations, an active engine mounting system based on smart materials, such as piezo elements, magnetorheological (MR) fluids, and shape memory alloys, has been widely researched and developed, as shown in Fig. 1. Presently, several ongoing studies have been devoted to active mounting systems.

Typically, lead zirconate titanate (PZT) actuators are utilized in active engine mounting systems, such as those used in vehicles, aircraft, and unmanned aerial vehicles (UAVs)^1–4^. The energy of the piezoelectric shunt damper acting on the thin plate is reduced by optimizing the size, number and composition of the piezoelectric patches^5^. However, as control stability issues arise when the active mounts use only PZT actuators, they are combined with other elements, such as rubber. Lee proposed a hybrid engine mount system with MR and piezo-stack mounts. Experimental results demonstrated that this engine mount system performed well at various frequency ranges^6^. Jiang implemented active vibration control for a plate structure through structure-actuator-interaction (SAI). The experimental results indicated that the stiffness effect of the plate structure could be increased, and the first and second modes could be controlled^7^. Mansour proposed an electromechanical actuator to solve the variable-displacement engine (VDE) isolation problem, and experimental results indicated that the designed electromechanical actuator could cope with complex vibration modes^8^. Shangguan presented a powertrain mounting system consisting of hydraulic engine mounts and calculation methods for determining powertrain displacements. The simulation results demonstrated that the bounce and roll modes and the reaction forces could be reduced^9^. Furthermore, several active control algorithms are widely utilized in active mounting systems, such as vehicles^10–15^, marine engines^16^, and hydraulic excavators^17^. Park proposed taking into consideration the non-proportional viscous damping in vehicle mounting systems and analyzed the influence of spectrally varying mount properties, including the effects of stiffness and damping^18,19^. Hu proposed a new analytical axiom for powertrain mounting systems to expand torque roll axis decoupling theories, and it showed a complete decoupling of the motions^20^.

Active vibration control based on an active mounting system exhibits superior performance. However, a significant cost is associated with controlling the actual engine, which becomes an obstacle to commercialization. In particular, as shown in Fig. 1, as vehicle mounting locations are usually designed without considering NVH performance, optimal vibration control cannot be expected even though active mounts are utilized. Thus, identifying the optimal position of the active mounting system is crucial to reducing the control cost. Several studies focused on the optimal position of an active engine mounting system to reduce vibrations. Shang proposed a modifed version of the Sine Cosine algorithm (MSCA) to solve the optimization problem. Compared with other algorithms, the algorithm has good convergence and robustness^21^. Sang proposed a meta-heuristic algorithm to solve optimization problems. Experiments results show that the computation time for complex optimization problems is very short^22^. Dehghani introduced a Hybrid Leader Optimization (HLBO) algorithm for optimization challenge. Compared with other algorithms, indicate the convergence of HLBO is faster in local search^23^. Nadi proposed a singular value decomposition method to find the optimal layout of piezoelectric actuators and sensors. The results show that the vibration reduction effect of the cantilever thick plate has been significantly improved^24^.Bruant proposed using a genetic algorithm to identify the optimal actuator and sensor locations that minimize mechanical energy and maximize the energy output of the state, respectively^25–27^. Ooi determined the optimal location and orientation angle of each engine mount using a dynamic optimization process to achieve minimum mean force transmissibility^28^. Hafidi developed an indirect method to measure the dynamic forces of a powertrain, and demonstrated an approach to identify the optimal position of engine mounts^29^. Kumar studied the vibration attributes of heavy commercial vehicles with respect to different engine mount positions. The results indicated that the engine isolator can be altered along the longitudinal direction of the powertrain^30^. Chhabra proposed a modified control matrix and singular value decomposition (MCSVD) approach using a modified heuristic genetic algorithm (MHGA) to consider the optimal placement of piezo-patches on a simply supported thin plate. The proposed algorithm exhibited superior performance when compared to the genetic algorithm^31^. Tham developed a smart nine-noded isoparametric element based on first-order shear deformation theory for optimal placement and active vibration control of laminated composite plates^32^. Hasheminejad proposed the optimal position of the piezoelectric actuator and sensor for a transversely isotropic cylindrical panel using a genetic algorithm-based optimal placement procedure. The proposed position demonstrated excellent vibration control performance when pulse load and white noise were used as the disturbance forces^33^. Biglar proposed a new formulation for the combined active vibration and optimal configuration of a plate structure to suggest the optimal location for piezoelectric actuators and sensors, and indicated that the damping effect could be increased and the amplitude of the plate reduced^34^. Liette accomplished vibration control by employing an active path consisting of piezo stack actuators and rubber mounts and quantifying the active path interaction for the dynamic and passive characteristics. The simulation and experimental results showed that vibration isolation can be achieved to some extent^35^. However, under the same excitation, the effect on vibration reduction can vary based on the differing locations of the active mounts.

## Problem formulation

The scope of this study is restricted to controlling the mass movement at the source from a source-path-receiver system with two vertical active structural pathways, and dealing with dynamic interactions through the paths in the mid-frequency range.

**Figure 2:**
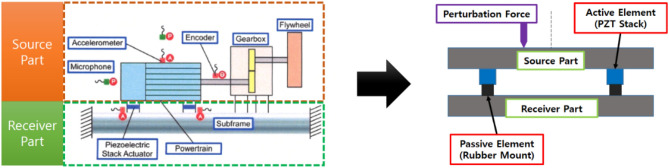
Active element integration for mitigating source movement from perturbation force.

A streamlined active structural system with two paths containing integrated active elements to mitigate the source movement from perturbation forces^36–40^ is investigated for analytical purposes, as shown in Fig. 2. In comparison to a powertrain of electric vehicles, the source part would be an electric motor, the paths would be active mounts with a rubber mount and an active element, and the receiver parts would be the vehicle sub-frame. The specific objectives are set as follows: (1) elaborate a mathematical model of the structure, (2) analyze the actuator amplitude and phase with the defined performance indication, (3) set mount positioning criteria for minimizing the secondary force from the active element actuator, and (4) design a feasibility experiment and validate the proposed position criteria. It is assumed that the system is deterministic, discrete, independent of frequency, and linear around a point of operation, without nonlinear kinematic effects. Consequently, the superposition principle is applicable, the movements are relatively small, and higher-order terms can be ignored. Additionally, each part is assumed to be rigid without flexural participation, and a known external harmonic perturbation force excites only the source part. Control algorithms are not applied, and the control force is derived analytically rather than being based on the behavior of the system in the steady state. In structural routes, passive components are assumed to be without mass, while the active components have a mass, and structural damping is assumed at each spring element with a constant damping coefficient. The structure has no motion except in the vertical direction, since its width is relatively small compared to its length. The active elements are piezoelectric (PZT) stack actuators.

## Modeling of active mounting system

### Lumped parameter model

**Figure 3:**
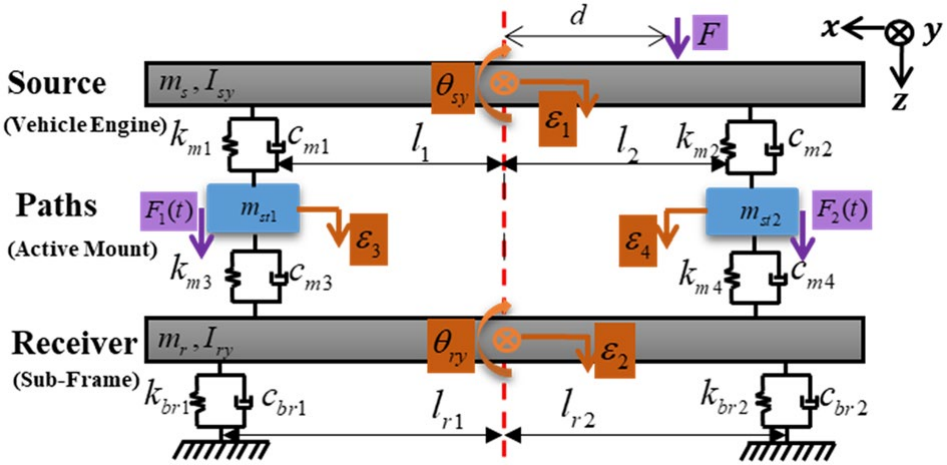
6-DOF lumped parameter model of given source-path-receiver system.

**Figure 4:**
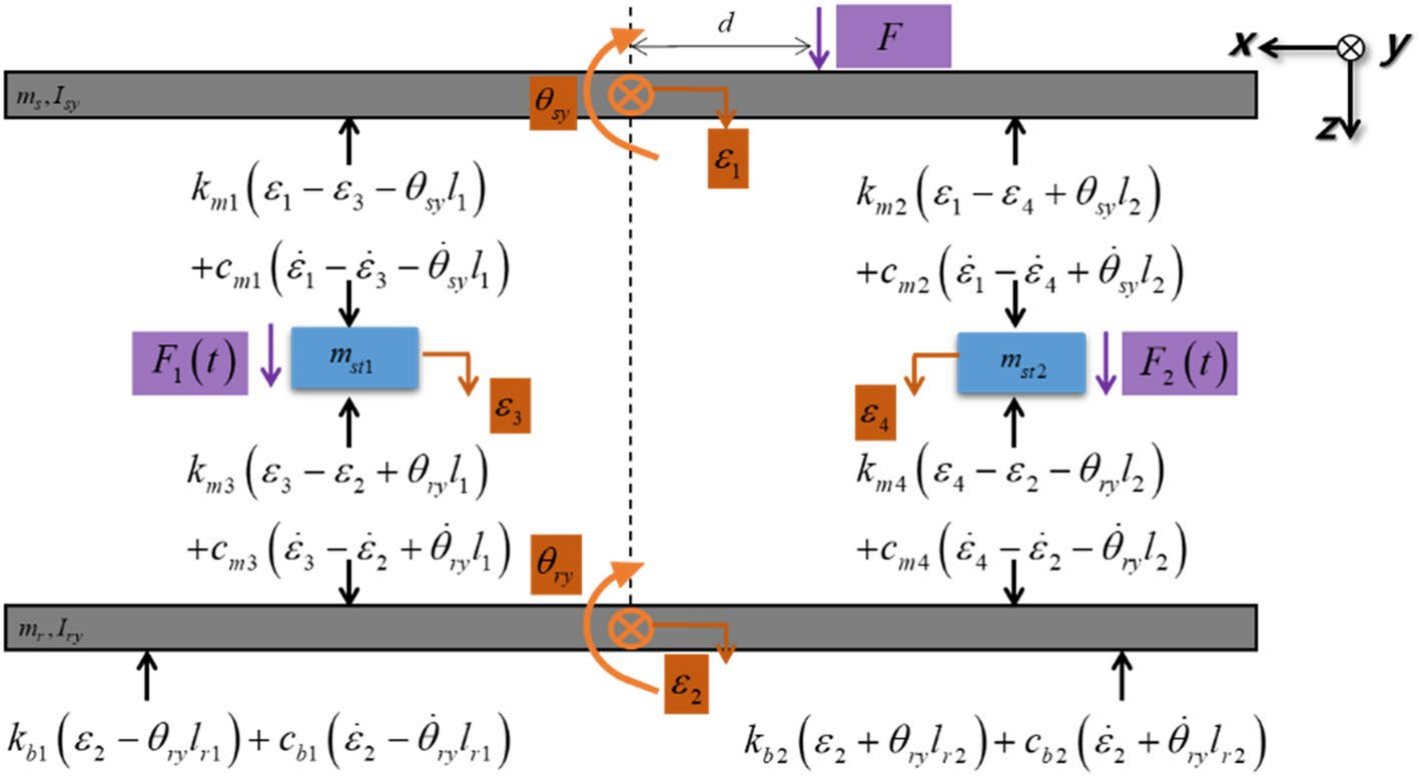
Free-body diagram of the system.

The overall model of a source-path-receiver system with two vertical active structural pathways was established based on the lumped parameter model, as shown in Fig. 3. Here, the source represents a powertrain (electric motor, engine, etc.), paths represent active mounts consisting of a piezoelectric stack actuator and a rubber mount to provide active vibration isolation, and the receiver represents the vehicle sub-frame. Additionally, the free-body diagram of the overall model is depicted in Fig. 4. In this study, it is assumed that displacement exists only in the z-direction, and rotational motion exists only in the y-direction. Thus, the overall model has four translational motions and two rotational motions, indicating that it is a 6 DOF system.

In Fig. 3, $${m}_{s}$$ and $${m}_{r}$$ are the source mass and receiver mass. $${m}_{st1}$$ and $${m}_{st2}$$ are the masses of the piezoelectric stack actuators. $${I}_{sy}$$ and $${I}_{ry}$$ represent the inertia of source and receiver (*y* refers to the Cartesian coordinate). $${\theta }_{sy}$$ is the rotational motion of source (*s* refers to source). $${\theta }_{ry}$$ is the rotational motion of receiver (*r* refers to receiver). $${l}_{i}$$ represent distance between the center of gravity and each actuator (*i* is a actuator index). $${l}_{rn}$$ are characteristic lengths of receiver (*n* is a mount index). $$d$$ represent the distance from the center of the gravity to the perturbation force. $$F$$ is the perturbation force. $${F}_{i}(t)$$ represents the required control force of each actuator. $${\varepsilon }_{1}$$ and $${\varepsilon }_{2}$$ are the displacement of source and receiver. $${\varepsilon }_{3}$$ and $${\varepsilon }_{4}$$ indicate the displacement of actuator 1 and actuator 2, respectively. $$i=\sqrt{-1}$$*,* $$k_{mi} = \,\,\overset{\lower0.5em\hbox{$\smash{\scriptscriptstyle\smile}$}}{k} \,_{mi} \,\left( {1 + i\eta_{mi} } \right)$$ are the path mount complex valued stiffness with $$\overset{\lower0.5em\hbox{$\smash{\scriptscriptstyle\smile}$}}{k}_{mi}$$ as the real part and $${\eta }_{mi}$$ as the loss factor. $${c}_{mi}$$ are the damping coefficients of the path mount. $$k_{bri} = \,\overset{\lower0.5em\hbox{$\smash{\scriptscriptstyle\smile}$}}{k}_{bri} \left( {1 + i\eta_{bri} } \right)$$ are the receiver mount complex valued stiffness with $$\overset{\lower0.5em\hbox{$\smash{\scriptscriptstyle\smile}$}}{k}_{bri}$$ as the real part and $${\eta }_{bri}$$ as the loss factor. $${c}_{bri}$$ are the damping coefficients of receiver mount.

The model parameter values are listed in Table 1. The masses and lengths are measured. The moments of inertia are calculated according to the length, width and height of the model. A chirp voltage signal applied to the actuator, and find out the resonant frequency of the actuator through experiments. $k={\omega }^{2}{m}_{st1}$, where $\omega$ is the natural frequency and ${m}_{st1}$ is the actuator mass. $\eta =({\omega }_{2}-{\omega }_{1})/\omega$, where ${\omega }_{1}$ and ${\omega }_{2}$ are the half-power frequencies. The values of ${k}_{m2}$, ${k}_{m3}$, ${k}_{m4}$, ${k}_{brn}$ are obtained in the same manner.

**Table 1:** Identified system parameters.

Parameter	Value	Units
${m}_{s}={m}_{r}$	$1.395$	$\mathrm{kg}$
${m}_{st1}={m}_{st2}$	$0.075$	$\mathrm{kg}$
${I}_{sy}={I}_{ry}$	$18.675$	${\mathrm{gm}}^{2}$
${k}_{m1}$	$5.64(1+\mathrm{i}0.036)$	${\mathrm{kN mm}}^{-1}$
${k}_{m2}$	$2.48(1+\mathrm{i}0.036)$	${\mathrm{kN mm}}^{-1}$
${k}_{m3}$	$0.61(1+\mathrm{i}0.300)$	${\mathrm{kN mm}}^{-1}$
${k}_{m4}$	$0.530(1+\mathrm{i}0.300)$	${\mathrm{kN mm}}^{-1}$
${k}_{brn}$	$0.420(1+\mathrm{i}0.300)$	${\mathrm{kN mm}}^{-1}$
$d$	$67$	$\mathrm{mm}$
${l}_{1}$	$67$	$\mathrm{mm}$
${l}_{2}$	$118$	$\mathrm{mm}$
${l}_{r1}={l}_{r2}$	$189$	$\mathrm{mm}$

Based on the Newton’s Second Law, the resulting equations of motion is expressed in the compact matrix form in center-of-mass coordinates as

1 $$M\ddot{q}\left(t\right)+C\dot{q}\left(t\right)+Kq\left(t\right)={F}_{A}\left(t\right)+{F}_{P}\left(t\right),$$

where q is the generalized displacement vector. *M* is the inertia matrix, *K* and* C* are the complex valued stiffness matrix and damping matrix. ${F}_{A}$ is the required control force vector and ${F}_{P}$ is the perturbation force vector. Here,

2 $$M=diag\left(\left\{\begin{array}{cccccc}{m}_{s}& {m}_{r}& {m}_{st1}& {m}_{st2}& {I}_{sy}& {I}_{ry}\end{array}\right\}\right),$$

3 $${F}_{P}={\left\{\begin{array}{cccccc}F& 0& 0& 0& F{d}_{1}& 0\end{array}\right\}}^{T},$$

4 $${F}_{A}={\left\{\begin{array}{cccccc}0& 0& {F}_{1}& {F}_{2}& 0& 0\end{array}\right\}}^{T},$$

5 $$q={\left\{\begin{array}{cccccc}{\varepsilon }_{1}& {\varepsilon }_{2}& {\varepsilon }_{3}& {\varepsilon }_{4}& {\theta }_{sy}& {\theta }_{ry}\end{array}\right\}}^{T},$$

6 $$K=\left[\begin{array}{cccccc}{k}_{m1}+{k}_{m2}& 0& -{k}_{m1}& -{k}_{m2}& {k}_{m2}{l}_{2}-{k}_{m1}{l}_{1}& 0\\ 0& {k}_{m3}+{k}_{m4}+{k}_{b1}+{k}_{b2}& -{k}_{m3}& -{k}_{m4}& 0& {k}_{m4}{l}_{2}-{k}_{m3}{l}_{1}+{k}_{b2}{l}_{r2}-{k}_{b1}{l}_{r1}\\ -{k}_{m1}& -{k}_{m3}& {k}_{m1}+{k}_{m3}& 0& {k}_{m1}{l}_{1}& {k}_{m3}{l}_{1}\\ -{k}_{m2}& -{k}_{m4}& 0& {k}_{m2}+{k}_{m4}& -{k}_{m2}{l}_{2}& -{k}_{m4}{l}_{2}\\ {k}_{m2}{l}_{2}-{k}_{m1}{l}_{1}& 0& {k}_{m1}{l}_{1}& -{k}_{m2}{l}_{2}& {k}_{m1}{l}_{1}^{2}+{k}_{m2}{l}_{2}^{2}& 0\\ 0& {k}_{m4}{l}_{2}-{k}_{m3}{l}_{1}+{k}_{b2}{l}_{r2}-{k}_{b1}{l}_{r1}& {k}_{m3}{l}_{1}& -{k}_{m4}{l}_{2}& 0& {k}_{m3}{l}_{1}^{2}+{k}_{m4}{l}_{2}^{2}+{k}_{b1}{l}_{r1}^{2}+{k}_{b2}{l}_{r2}^{2}\end{array}\right],$$

7 $$C=\left[\begin{array}{cccccc}{c}_{m1}+{c}_{m2}& 0& -{c}_{m1}& -{c}_{m2}& {c}_{m2}{l}_{2}-{c}_{m1}{l}_{1}& 0\\ 0& {c}_{m3}+{c}_{m4}+{c}_{b1}+{c}_{b2}& -{c}_{m3}& -{c}_{m4}& 0& {c}_{m4}{l}_{2}-{c}_{m3}{l}_{1}+{c}_{b2}{l}_{r2}-{c}_{b1}{l}_{r1}\\ -{c}_{m1}& -{c}_{m3}& {c}_{m1}+{c}_{m3}& 0& {c}_{m1}{l}_{1}& {c}_{m3}{l}_{1}\\ -{c}_{m2}& -{c}_{m4}& 0& {c}_{m2}+{c}_{m4}& -{c}_{m2}{l}_{2}& -{c}_{m4}{l}_{2}\\ {c}_{m2}{l}_{2}-{c}_{m1}{l}_{1}& 0& {c}_{m1}{l}_{1}& -{c}_{m2}{l}_{2}& {c}_{m1}{l}_{1}^{2}+{c}_{m2}{l}_{2}^{2}& 0\\ 0& {c}_{m4}{l}_{2}-{c}_{m3}{l}_{1}+{c}_{b2}{l}_{r2}-{c}_{b1}{l}_{r1}& {c}_{m3}{l}_{1}& -{c}_{m4}{l}_{2}& 0& {c}_{m3}{l}_{1}^{2}+{c}_{m4}{l}_{2}^{2}+{c}_{b1}{l}_{r1}^{2}+{c}_{b2}{l}_{r2}^{2}\end{array}\right],$$

**Figure 5:**
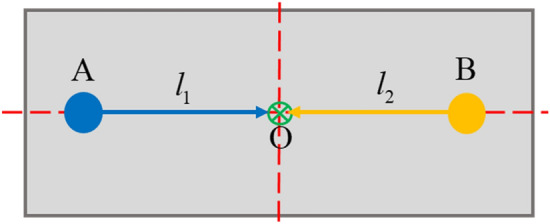
Positional relationship between actuators 1 and 2 (Top view of the system).

The above equation of motion was established considering the center-of-mass coordinates. As this study focuses on mitigating source movement from perturbation forces at active mount positions, coordinates should be transformed from the center-of-mass to the location of the active mounts. The relationship between the center-of-mass and each active mount is depicted in Fig. 5 and represented as Eq. (8). In Fig. 5, *A*, *B*, and *O* represent the positions corresponding to actuators 1 and 2 and the center of mass, respectively.

The original coordinates are provided by Eq. (5), and the new coordinates are defined in Eq. (8). To obtain the relationship between the two coordinates, a linear interpolation methodology was utilized, as described in Eq. (9).

8 $${q}^{*}\left(t\right)={\left\{\begin{array}{cccccc}{\xi }_{st1,g1}^{*}& {\xi }_{st2,g1}^{*}& {\varepsilon }_{3}& {\varepsilon }_{4}& {\xi }_{st1,g2}^{*}& {\xi }_{st2,g2}^{*}\end{array}\right\}}^{T},$$

9 $$O=\frac{{l}_{2}}{{l}_{1}+{l}_{2}}A+\frac{{l}_{1}}{{l}_{1}+{l}_{2}}B.$$

Using Eq. (9) and other similar relationships, the transformation matrix can be obtained as in Eq. (10).

10 $${O}^{*}=\left[\begin{array}{cccccc}\frac{{l}_{2}}{{l}_{1}+{l}_{2}}& \frac{{l}_{1}}{{l}_{1}+{l}_{2}}& 0& 0& 0& 0\\ 0& 0& 0& 0& \frac{{l}_{r2}}{{l}_{r1}+{l}_{r2}}& \frac{{l}_{r1}}{{l}_{r1}+{l}_{r2}}\\ 0& 0& 1& 0& 0& 0\\ 0& 0& 0& 1& 0& 0\\ \frac{-1}{{l}_{1}+{l}_{2}}& \frac{1}{{l}_{1}+{l}_{2}}& 0& 0& 0& 0\\ 0& 0& 0& 0& \frac{-1}{{l}_{r1}+{l}_{r2}}& \frac{1}{{l}_{r1}+{l}_{r2}}\end{array}\right].$$

**Figure 6:**
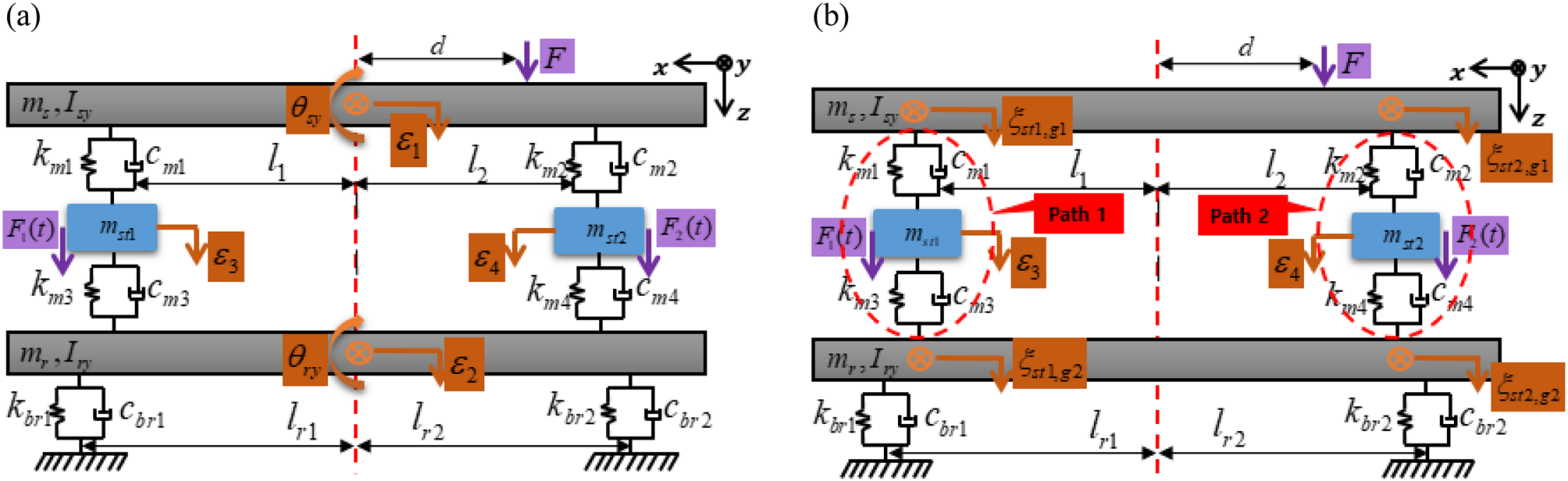
Schematic representation: (**a**) Coordinates at center of mass and (**b**) Coordinates at active path.

Applying Eq. (10), the coordinate transform is performed, and it is defined as $q={O}^{*}{q}^{*}$, where ${q}^{*}$ is defined in Eq. (10). Thus, in order to consider the transformed coordinates, Eq. (1) is re-written in relation to the active mount coordinates, defined in Eq. (11), where ${M}^{*}=M{O}^{*}$, ${C}^{*}=C{O}^{*}$, and ${K}^{*}=K{O}^{*}$. Furthermore, Fig. 6 shows the transformed coordinates.

11 $${M}^{*}{\ddot{q}}^{*}\left(t\right)+{C}^{*}{\dot{q}}^{*}\left(t\right)+{K}^{*}{q}^{*}\left(t\right)={F}_{A}\left(t\right)+{F}_{P}\left(t\right),$$

### Control force and phase for vibration isolation

The relative phase between the harmonic force and system motion plays an important role when considering the relationship between them. To manage the phase along with the amplitude, the disturbance and control forces are assumed to be complex values. It is defined through Eqs. (12) and (13).

12 $$F\left(t\right)={W}_{1}{e}^{i\omega t},$$

13 $${F}_{i}^{*}\left(t\right)={F}_{i}{e}^{i\left(\omega t+{\varnothing }_{sti}\right)},$$

where ${W}_{1}$ and ${F}_{i}$ represent the amplitudes corresponding to the shaker and the *ith* actuator, respectively. ${\phi }_{sti}$ represents the phase corresponding to the *ith* actuator. To simplify the notation, the displacement of each active path is rewritten using Eq. (14).

14 $${\xi }_{i}^{*}\left(t\right)={\xi }_{sti, g1}^{*}\left(t\right).$$

To isolate the vibration, ${\xi }_{i}^{*}$ should be minimized. Considering that the given system is linear, the source mass movement ${\xi }_{i}^{*}$ can be defined using Eq. (15).

15 $${\xi }_{i}^{*}\left(t\right)=\left({\Xi }_{si,1}^{*}+{\Xi }_{\text{si,st1}}^{*}{e}^{i{\phi }_{st1}}+{\Xi }_{\text{si,st2}}^{*}{e}^{i{\phi }_{st2}}\right){e}^{i\omega t}.$$

In Eq. (15), ${\Xi }_{si,1}^{*}$ represents the complex amplitude of the *ith* path corresponding to the disturbance force. ${\Xi }_{si,st1}^{*}$ and ${\Xi }_{si,st2}^{*}$ represent the complex amplitudes corresponding to actuator forces, respectively. To calculate the amplitude and phase, the compliance matrix ${H}^{*{^{\prime}}}$ is defined as in Eq. (16).

16 $${H}^{*{^{\prime}}}=\left[\begin{array}{cccccc}{H}_{11}^{*{^{\prime}}}& {H}_{12}^{*{^{\prime}}}& {H}_{13}^{*{^{\prime}}}& {H}_{14}^{*{^{\prime}}}& {H}_{15}^{*{^{\prime}}}& {H}_{16}^{*{^{\prime}}}\\ {H}_{21}^{*{^{\prime}}}& {H}_{22}^{*{^{\prime}}}& {H}_{23}^{*{^{\prime}}}& {H}_{24}^{*{^{\prime}}}& {H}_{25}^{*{^{\prime}}}& {H}_{26}^{*{^{\prime}}}\\ {H}_{31}^{*{^{\prime}}}& {H}_{32}^{*{^{\prime}}}& {H}_{33}^{*{^{\prime}}}& {H}_{34}^{*{^{\prime}}}& {H}_{35}^{*{^{\prime}}}& {H}_{36}^{*{^{\prime}}}\\ {H}_{41}^{*{^{\prime}}}& {H}_{42}^{*{^{\prime}}}& {H}_{43}^{*{^{\prime}}}& {H}_{44}^{*{^{\prime}}}& {H}_{45}^{*{^{\prime}}}& {H}_{46}^{*{^{\prime}}}\\ {H}_{51}^{*{^{\prime}}}& {H}_{52}^{*{^{\prime}}}& {H}_{53}^{*{^{\prime}}}& {H}_{54}^{*{^{\prime}}}& {H}_{55}^{*{^{\prime}}}& {H}_{56}^{*{^{\prime}}}\\ {H}_{61}^{*{^{\prime}}}& {H}_{62}^{*{^{\prime}}}& {H}_{63}^{*{^{\prime}}}& {H}_{64}^{*{^{\prime}}}& {H}_{65}^{*{^{\prime}}}& {H}_{66}^{*{^{\prime}}}\end{array}\right].$$

Using the compliance matrix, the magnitude and phase generated from the active path are defined as in Eqs. (17) and (18).

17 $${\Xi }_{\text{si,1}}^{*}=\left({H}_{\text{i1}}^{*^{\prime}}\text{+}{\text{H}}_{\text{i5}}^{*^{\prime}}d\right){W}_{1}, {\Xi }_{\text{si,stj}}^{*}\text{=}{\text{H}}_{\text{i3}}^{*^{\prime}}{F}_{j},$$

18 $${\beta }_{\text{si,1}}=\angle \left({H}_{\text{i1}}^{*^{\prime}}\text{+}{\text{H}}_{\text{i5}}^{*^{\prime}}d\right), {\beta }_{\text{si,stj}}=\angle {H}_{\text{i3}}^{*^{\prime}}.$$

Equations (17) and (18) represent the magnitude and phase associated with the *ith* active path, respectively. Additionally, $\angle$ stands for the phase operator. ${\beta }_{si,1}$ represents the phase generated by the disturbance force on the *ith* path. ${\beta }_{si,stj}$ represents the phase generated by the *jth* actuator along the *ith* path. Equation (15) is rewritten in terms of magnitude and phase as Eq. (19).

19 $${\xi }_{i}^{*}\left(t\right)=\left(\left|{\Xi }_{\text{si,1}}^{*}\right|{e}^{i{\beta }_{si,1}}+\left|{\Xi }_{\text{si,st1}}^{*}\right|{e}^{i\left({\beta }_{si,st1}+{\phi }_{st1}\right)}+\left|{\Xi }_{\text{si,st2}}^{*}\right|{e}^{i\left({\beta }_{si,st2}+{\phi }_{st2}\right)}\right){e}^{i\omega t}.$$

In Eq. (19), there are several phase terms. Thus, to perform motion control, the phase is assumed to be generated by the disturbance force. Based on this assumption, it is possible to create out-of-phase motion. To match the phase, ${\phi }_{sti}$ is defined as in Eq. (20).

20 $${\phi }_{\text{sti}}={\beta }_{\text{si,1}}-{\beta }_{\text{si,stj}}.$$

Using Eqs. (19) and (20) can be rewritten as Eq. (21).

21 $${\xi }_{i}^{*}\left(t\right)=\left(\left|{\Xi }_{\text{si,1}}^{*}\right|+\left|{\Xi }_{\text{si,st1}}^{*}\right|+\left|{\Xi }_{\text{si,st2}}^{*}\right|\right){e}^{i(\omega t+{\beta }_{si,1})}.$$

To reduce the source motion to zero, the actuator forces are calculated by assuming that the magnitude is zero in Eq. (21). Hence, magnitude in ${\xi }_{i}^{*}$ can be defined as in Eq. (22a, b).

$${\xi }_{st1,g1}^{*}=\left|{\Xi }_{s\mathrm{1,1}}^{{*}^{^{\prime}}}\right|+\left|{\Xi }_{s\mathrm{1,3}}^{{*}^{^{\prime}}}\right|+\left|{\Xi }_{s\mathrm{1,4}}^{{*}^{^{\prime}}}\right|=0,$$

22a $$\Rightarrow F\left|{H}_{11}^{{*}^{^{\prime}}}+{H}_{15}^{{*}^{^{\prime}}}d\right|+{F}_{1}\left|{H}_{13}^{{*}^{^{\prime}}}\right|+{F}_{2}\left|{H}_{14}^{{*}^{^{\prime}}}\right|=0,$$

$${\xi }_{st2,g1}^{*}=\left|{\Xi }_{s\mathrm{2,1}}^{{*}^{^{\prime}}}\right|+\left|{\Xi }_{s\mathrm{2,3}}^{{*}^{^{\prime}}}\right|+\left|{\Xi }_{s\mathrm{2,4}}^{{*}^{^{\prime}}}\right|=0,$$

22b $$\Rightarrow F\left|{H}_{21}^{{*}^{^{\prime}}}+{H}_{25}^{{*}^{^{\prime}}}d\right|+{F}_{1}\left|{H}_{23}^{{*}^{^{\prime}}}\right|+{F}_{2}\left|{H}_{24}^{{*}^{^{\prime}}}\right|=0,$$

Using Eq. (22a, b), the actuator force can be calculated as shown in Eq. (23).

23 $$\left\{\begin{array}{c}{F}_{1}\\ {F}_{2}\end{array}\right\}=\frac{F}{\left|{H}_{13}^{{*}^{^{\prime}}}\right|\left|{H}_{24}^{{*}^{^{\prime}}}\right|-\left|{H}_{14}^{{*}^{^{\prime}}}\right|\left|{H}_{23}^{{*}^{^{\prime}}}\right|}\left\{\begin{array}{c}\left|{H}_{14}^{{*}^{^{\prime}}}\right|\left|{H}_{21}^{{*}^{^{\prime}}}+ \text{d} {\text{H}}_{25}^{{*}^{^{\prime}}}\right|-\left|{H}_{24}^{{*}^{^{\prime}}}\right|\left|{H}_{11}^{{*}^{^{\prime}}}+ \text{d} {\text{H}}_{15}^{{*}^{^{\prime}}}\right|\\ \left|{H}_{23}^{{*}^{^{\prime}}}\right|\left|{H}_{11}^{{*}^{^{\prime}}}+ \text{d} {\text{H}}_{15}^{{*}^{^{\prime}}}\right|-\left|{H}_{13}^{{*}^{^{\prime}}}\right|\left|{H}_{21}^{{*}^{^{\prime}}}+ \text{d} {\text{H}}_{25}^{{*}^{^{\prime}}}\right|\end{array}\right\}.$$

## Analytical approaches for determining positioning criteria

### One-way variation of active path locations

This section describes the dynamic and static analyses performed to determine the optimal location of the actuators. It is desirable for the actuators to have the lowest possible control force, given that a low control force guarantees a greater gain variation for the actuator, thereby maximizing the vibration reduction.

#### Case I: changing the position of actuator 1

**Figure 7:**
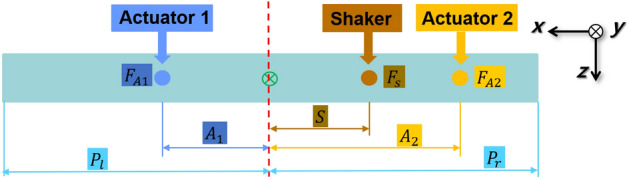
Positions of shaker, actuator 1, and actuator 2.

**Figure 8:**
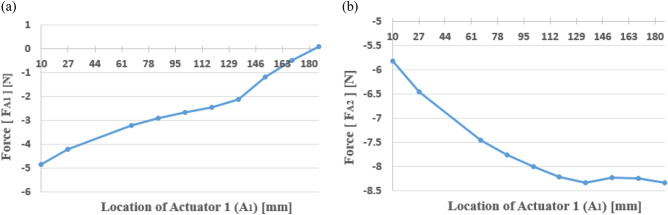
Actuator force of each path according to the location of actuator 1 (A1): (**a**) path 1; (**b**) path 2.

First, a series of simulations were performed by changing the location of actuator 1 from the center to the end of the beam. Figure 7 shows the positions of the shaker and actuators 1 and 2. Here, ${F}_{A1}, {F}_{A2},$ and ${F}_{S}$ represent the secondary force from actuators 1 and 2, and the shaker force, respectively. ${A}_{1}$ and ${A}_{2}$ represent the distance from the center-of-mass to each path, respectively, and $S$ corresponds to the distance from the center-of-mass to the shaker. The required force of both actuators for all cases was calculated using Eq. (23) presented in Sect. “Control force and phase for vibration isolation” with the shaker and actuator 2 fixed at $S=50 mm$ and ${A}_{2} = 186 mm$. Actuator 1 was moved from the center to the left end of the beam; thus, $0<{A}_{1}<186 mm$, and the shaker force ${F}_{s}$ was set to 10 N. The forces of both actuators are plotted with respect to the location of actuator 1 as depicted in Fig. 8.

Fig. 8 shows that the control force required for actuator 2 is greater than the control force for actuator 1 and that the control force in the same direction is required for both actuators, indicating that they possess opposite directions with respect to the shaker force. When the vibration attenuation or isolation is accomplished after the control and the vibration level ideally becomes zero, the condition of the overall system can be assumed to be a type of motion equilibrium, and thus statics can be applied.

Initially, if the distance between the center and actuator 1 increases, the control force in actuator 1 will decrease considering the moment equilibrium about the y-axis. Moreover, to establish the moment equilibrium as well as neutralize the z-direction force from the shaker, actuator 2, which is located closer to the shaker than actuator 1, should have a relatively greater force than actuator 1.

To methodically validate the aforementioned assumptions, a static analysis was performed on the given model. Two equations can be derived based on the force equilibrium and moment equilibrium at the center of the beam structure. Eq. (24) is the equation for the force equilibrium, and Eq. (25) is the equation for the moment equilibrium. Substituting Eq. (24) into Eq. (25), Eqs. (26a, b) can be obtained.

24 $${F}_{s}+{F}_{A1}+{F}_{A2}=0,$$

25 $${F}_{s}S+{F}_{A2}{A}_{2}-{F}_{A1}{A}_{1}=0,$$

26a $${F}_{s}S+\left(-{F}_{s}-{F}_{A1}\right){A}_{2}-{F}_{A1}{A}_{1}=0\to {F}_{A1}=\left(\frac{S-{A}_{2}}{{A}_{1}+{A}_{2}}\right){F}_{s}=\left(-1+\frac{{A}_{1}+S}{{A}_{1}+{A}_{2}}\right){F}_{s}{F}_{s},$$

26b $${F}_{s}S+{F}_{A2}{A}_{2}-\left(-{F}_{s}-{F}_{A2}\right){A}_{1}=0 \to {F}_{A2}=\left(\frac{-{A}_{1}-S}{{A}_{1}+{A}_{2}}\right){F}_{s}=\left(-1+\frac{{A}_{2}-S}{{A}_{1}+{A}_{2}}\right){F}_{s},$$

**Figure 9:**
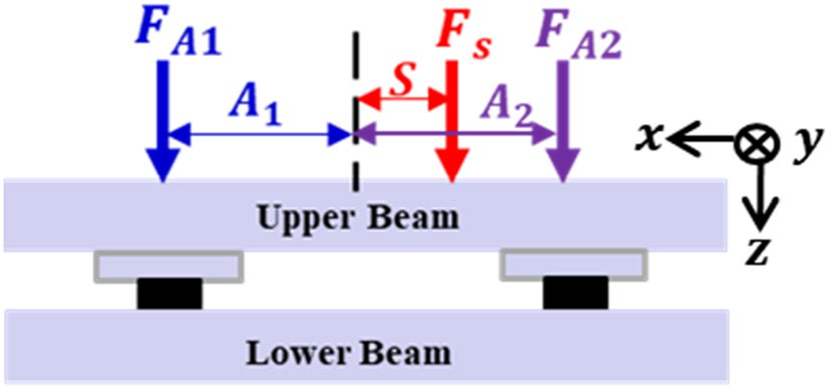
Free-body diagram for static analysis.

Subsequently, the required force for each actuator is plotted as the position of actuator 1 changes, as depicted in Fig. 9.

**Figure 10:**
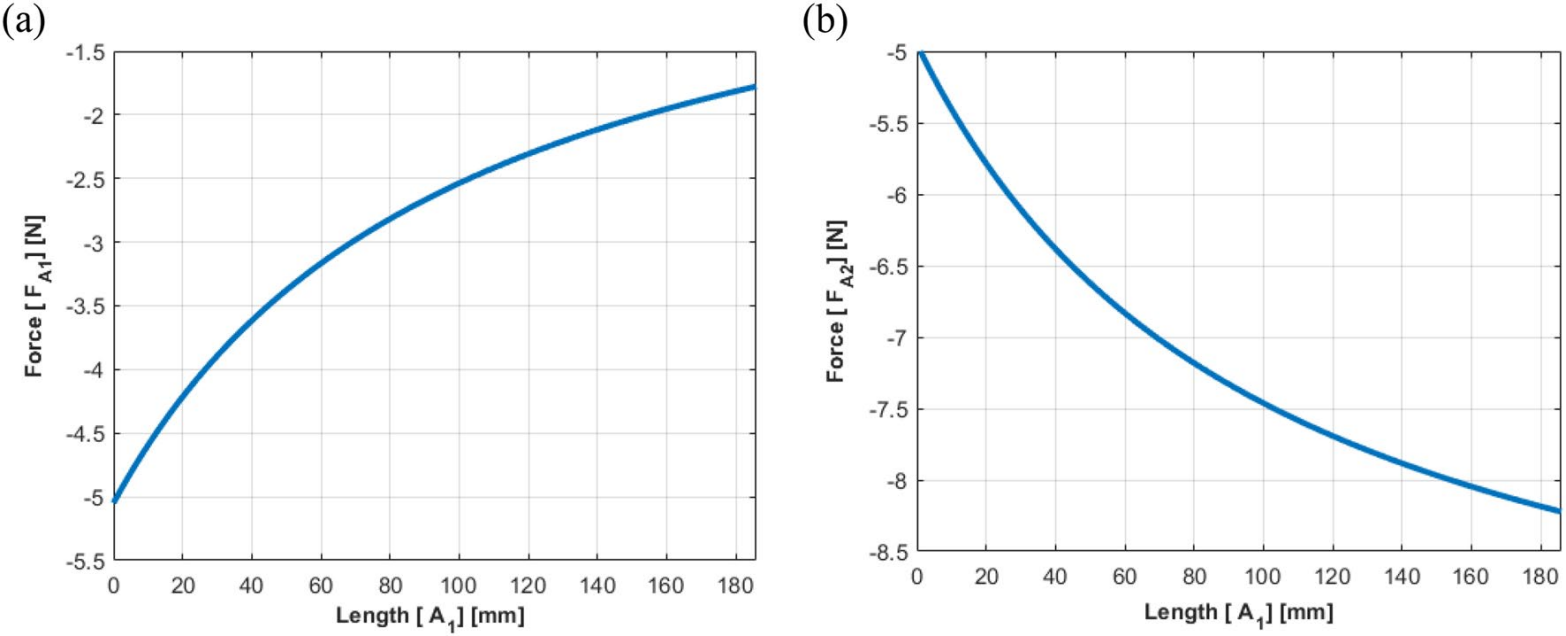
Actuator force estimated from the static equilibrium (case I): (**a**) path 1; (**b**) path 2.

In Fig. 10, the results show that ${F}_{A1}$ decreases with an increase in ${A}_{1}$, and ${F}_{A2}$ increases with an increase in ${A}_{1}$, which is consistent with the simulation results.

#### Case II: changing the position of actuator 2

**Figure 11:**
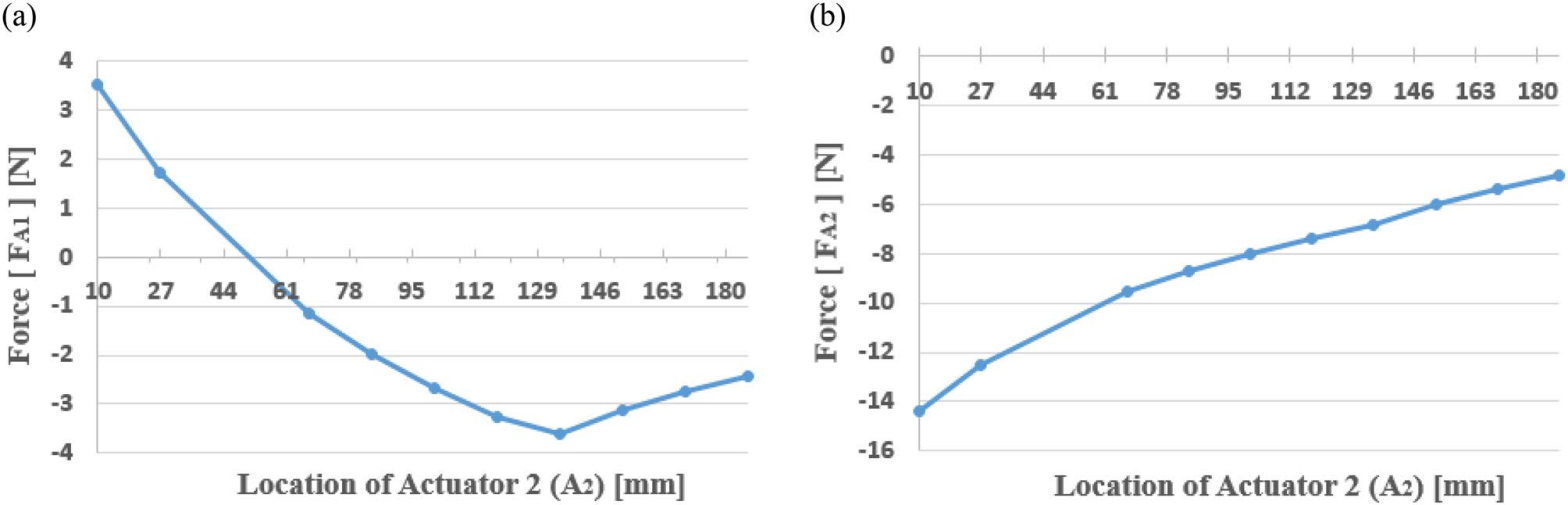
Actuator force of each path according to the location of actuator 2 (A2): (**a**) path 1; (**b**) path 2.

To confirm the required force of each actuator based on the change in the position of path 2, the actuator force was calculated using Eq. (23) presented in Sect. “Control force and phase for vibration isolation”. When the actuator force was calculated, the shaker and actuator 1 were fixed at $S=50 mm$ and ${A}_{1} = 101 mm$, respectively. Actuator 2 was moved from the center to the right end of the beam, such that $0<{A}_{2}<186 mm$. Furthermore, the shaker force ${F}_{s}$ was set to 10 N. Then, through simulation, the actuator force was plotted with respect to the location of actuator 2, the results of which are shown in Fig. 11.

Fig. 11 shows that when actuator 2 is placed on the left of the shaker, positive force is needed for actuator 1 and the absolute value of the force decreases. In contrast, when the actuator is placed on the right of the shaker, negative force is needed for actuator 1 and the absolute value of the force increases. When actuator 2 is placed on the left of the shaker, the two actuators are simultaneously located on the left of the shaker. In this case, since the force of the two actuators has different directions (the force for actuator 2 always has a negative sign, as shown in the right pane of Fig. 11), the clockwise movement about the center is inevitable. Thus, to attain static equilibrium, the two actuator forces should move in the same direction. Furthermore, greater control force is needed for actuator 2 when compared to that required for actuator 1, as actuator 2 moves to the right. Accordingly, it can be assumed that as actuator 2 is closer to the shaker, the required force is greater than that required for actuator 1.

**Figure 12:**
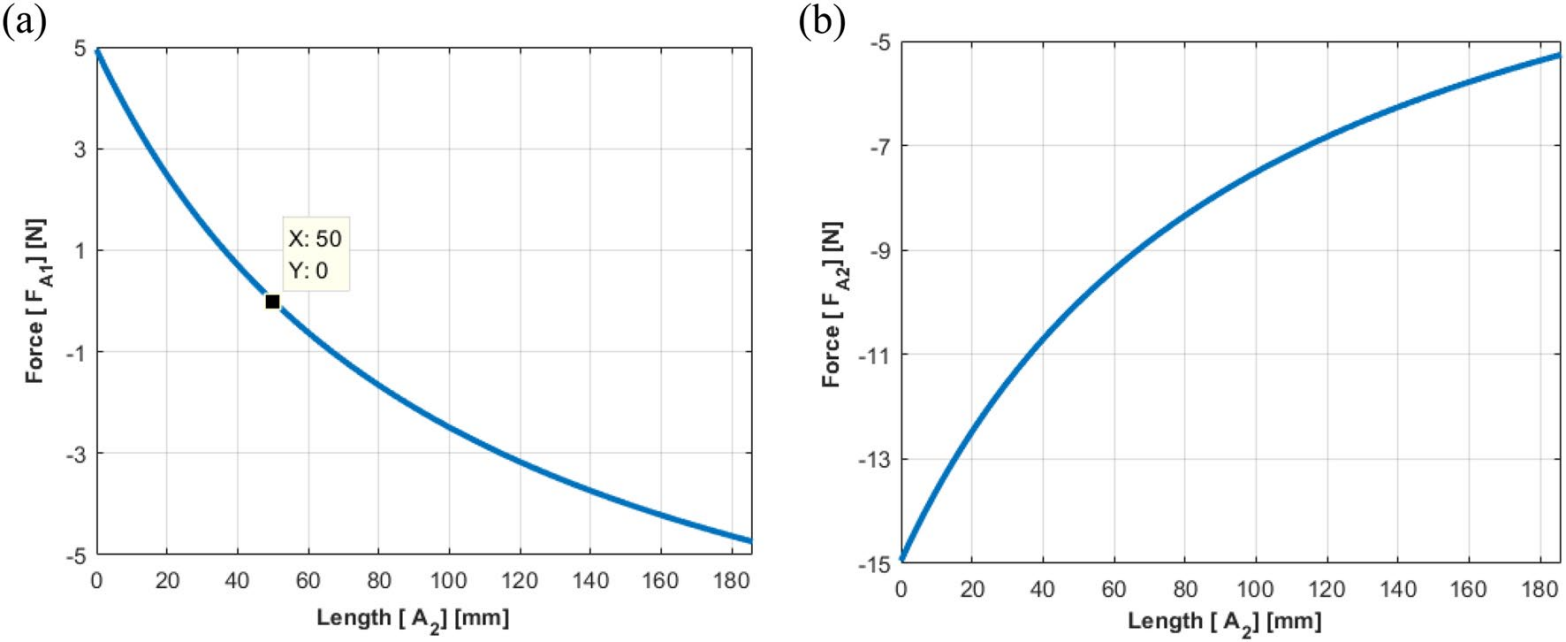
Actuator force estimated from the static equilibrium (Case II): (**a**) path 1; (**b**) path 2.

The results of the static analysis are shown in Fig. 12, where Eq. (26a, b) was applied while changing the location of actuator 2. While ${F}_{A1}$ tended to decrease and then increase with an increase in ${A}_{2}$, ${F}_{A2}$ decreased with an increase in ${A}_{2}$. Moreover, the plot crosses the x-axis near ${A}_{2}=50 mm$, indicating that actuator 2 is placed at the same position as the shaker. It can be concluded that the results exhibit the same trend as the simulation results.

Based on the above analysis, some guidelines regarding the location of the actuators can be outlined as follows: (1) as actuator 1 moves to the left of the beam, a smaller control force for actuator 1 and a greater control force for actuator 2 are required; (2) a smaller control force is needed in actuator 1, when actuator 2 is near the shaker; and (3) a greater control force is essential for actuator 2, as actuator 2 moves to the right of the beam. These conditions seem to be a trade-off for vibration control, considering the interaction between actuators 1 and 2. With the above approaches, it is infeasible to determine the optimal requirements for both actuators (minimizing the control force). Given that not all possible scenarios can be covered with them, the criteria for the optimal location could not be determined while the forces applied to each actuator and their effects were investigated. However, this shortcoming can be overcome by considering all possible locations for both the actuators.

### Two-way variation of active path locations

Figure 14 shows the positions of the shaker and actuators 1 and 2, where actuators 1 and 2 are located on either side of the beam and the shaker is installed on the right side of the beam. To analyze the actuator force, a series of simulations were conducted by changing the positions of the actuators as well as the shaker. The domains of the actuators and shaker were as follows: (1) the positions of actuators 1 and 2 are within the ranges of $21\le {A}_{1}\le {P}_{l}$ and $21\le {A}_{2}\le {P}_{r}$, respectively; (2) the shaker is located at points $S=40 mm$, $70 mm$, and $110 mm$.

#### Dynamic analysis

Based on the positions of the actuators, the control forces in each actuator can be calculated and analyzed using Eq. (23) from Sect. “Control force and phase for vibration isolation”. To observe the effects of changing the shaker position, three distinct scenarios were considered.

**Figure 13:**
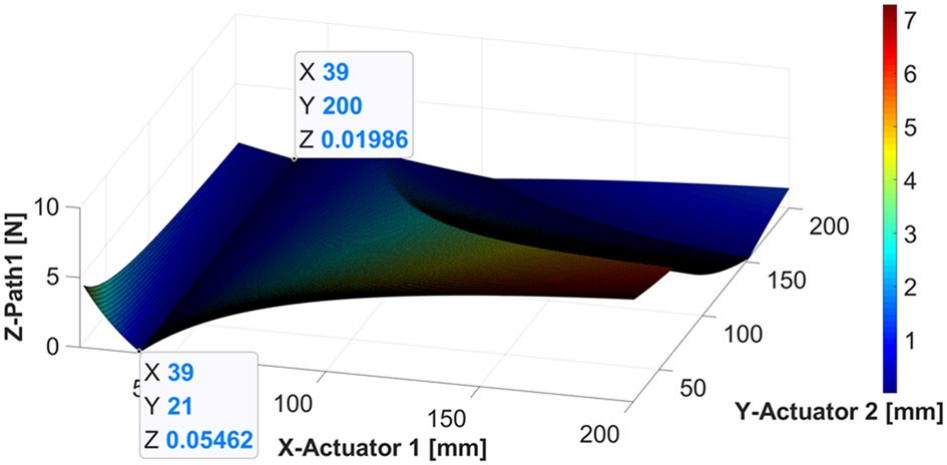
Control force of actuator 1 when the shaker is placed at 40 mm (*generated with MATLAB R2018b,* https://mathworks.com).

**Figure 14:**
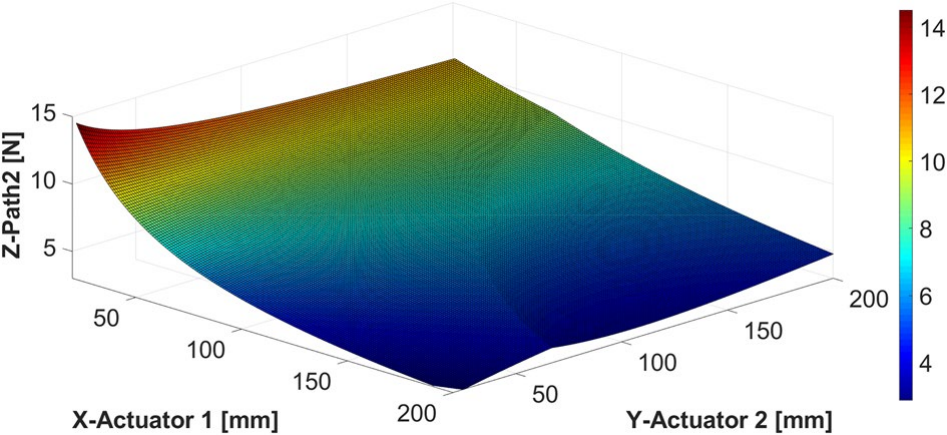
Control force of actuator 2 when the shaker is placed at 40 mm (*generated with MATLAB R2018b,* https://mathworks.com).

The results from the simulation when the shaker is located at 40 mm are shown in Figs. 13 and 14, depicting the force corresponding to the changing location of each actuator.

As shown in Fig. 13, the required control force of actuator 1 tends to first decrease and then increase as the position of actuator 1 moves from the center of gravity to the end of the beam. Based on these results, when actuator 1 is located at 39 mm (such that it is almost symmetric to the shaker position), the required force is minimized when compared with the other locations, regardless of the location of actuator 2. This implies that the effect of control is better than that at other positions. The required control force of actuator 1 decreased as actuator 2 moved to the end of the beam.

In Fig. 14, when actuator 1 is close to the end of the beam, the force of actuator 2 decreases, regardless of the position of actuator 2. Given that actuator 2 and the shaker are on the same side, the relative distance is shorter than the distance between actuator 1 and the shaker. Thus, it can be expected that the required control force on actuator 2 will be greater than that on actuator 1. Considering the size and performance of the piezoelectric stack actuator, actuator 2 is easier to control than actuator 1, and the size of the actuator should be determined based on the force required for actuator 2.

**Figure 15:**
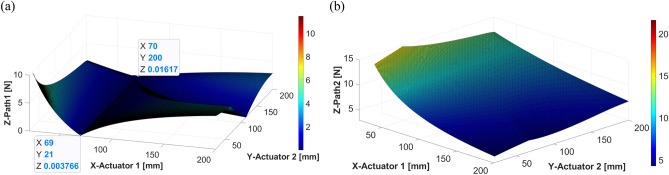
Control force of actuators 1 and 2 when the shaker is placed at 70 mm: (**a**) actuator 1; (**b**) actuator 2 (*generated with MATLAB R2018b,* https://mathworks.com).

The simulation results when the shaker is located at 70 mm are shown in Fig. 15, depicting the force corresponding to the changing location of each actuator. Similar to the previous case, when actuator 1 is placed at 70 mm (symmetric to the shaker position), the required force is minimal. Moreover, the above result is obtained regardless of the position of actuator 2. In addition, the required control force of actuator 1 decreases as actuator 1 moves to the end of the beam, and this trend is similar to the result of the static analysis. The control force of actuator 2 also exhibited a trend similar to that of the previous case.

It can be observed in Fig. 15 that regardless of the position of actuator 2, the force of actuator 2 decreases as actuator 1 approaches the end of the beam. As actuator 2 is placed on the same side as the shaker, the required control force on actuator 2 is much greater than that required on actuator 1.

**Figure 16:**
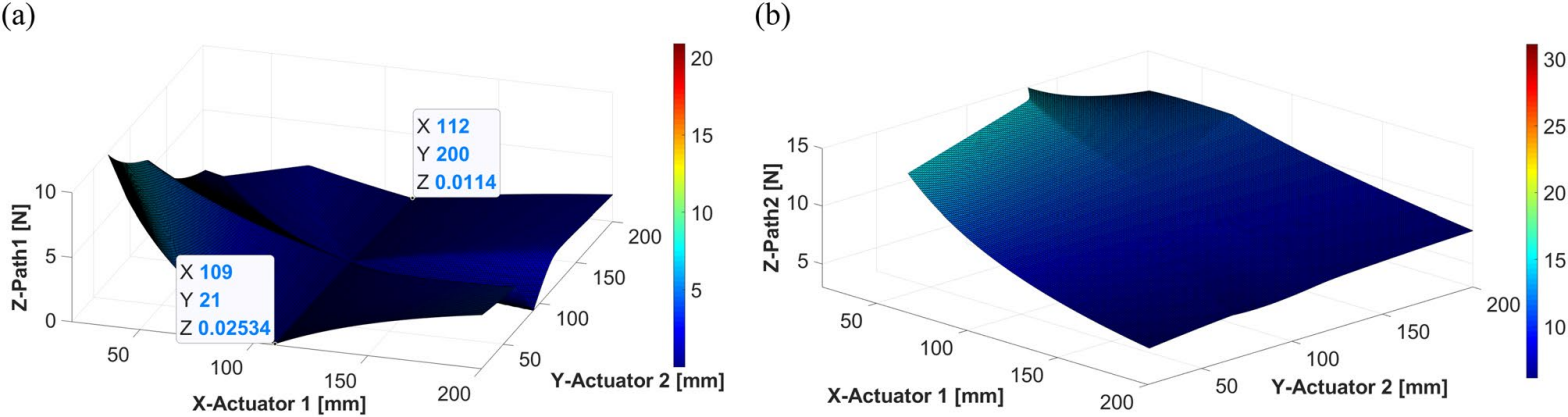
Control force of actuators 1 and 2 when the shaker is placed at 110 mm: (**a**) actuator 1; (**b**) actuator 2 (*generated with MATLAB R2018b,* https://mathworks.com).

The simulation results when the shaker is located at 110 mm are shown in Fig. 16, depicting the force corresponding to the changing location of each actuator. A similar trend was observed when the shaker was placed 40 and 70 mm away from the center of gravity of the beam. Thus, the results of the dynamic analysis can be summarized as follows: (1) actuator 1 is placed symmetrical to the shaker to increase the effect of control, (2) the control force of actuator 2 is diminished when actuator 1 moves away from the center of gravity toward the edge, and (3) the actuator specifications will be determined based on the force required for actuator 2, as greater force is required on actuator 2 than that on actuator 1. These trends are identical to those from the static analysis.

As shown in Table 2, when actuator 1 is located away from the center of gravity, the control force of actuator 1 is similar to a valley line, first decreasing and then increasing, and the valley is at 67 mm. This position corresponds to the symmetry of the shaker. Moreover, it is evident that the control force of actuator 2 diminishes, irrespective of the changes to the position of actuator 2.

**Table 2:** Control forces needed on actuators.

Shaker	Actuator 1	${F}_{1}$[N]	Actuator 2	${F}_{2}$[N]
67 mm	27 mm	2.662	118 mm	12.760
	50 mm	0.940		11.030
	67 mm	0.057		10.040
	84 mm	0.887		9.211
	101 mm	1.588		8.515
	118 mm	1.867		7.841
	135 mm	1.360		7.079
	152 mm	0.939		6.447
	169 mm	0.582		5.916
	186 mm	0.274		5.466

#### Static analysis

To compare the results from the dynamic analysis in Section “Dynamic analysis” with the results from static analysis, the static analysis was performed using the secondary force equation defined in Section “Analytical approaches for determining positioning criteria”. Thus, the simulation was conducted using conditions identical to those of the dynamic analysis. Additionally, an arbitrary location was selected, and an analysis was performed to validate the simulation results.

**Figure 17:**
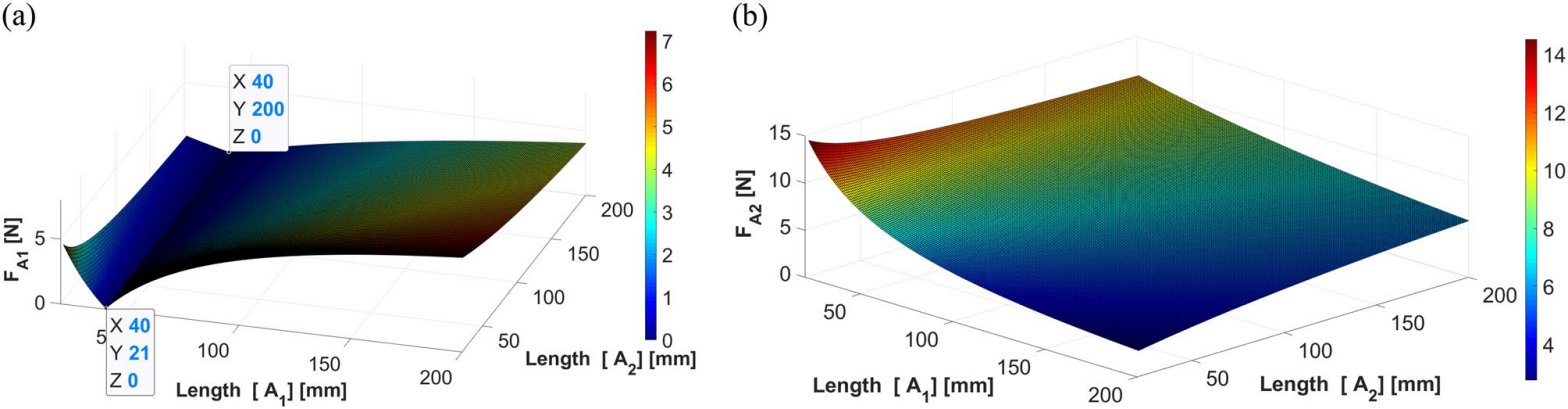
Control force of actuators 1 and 2 from static analysis (shaker at 40 mm): (**a**) actuator 1; (**b**) actuator 2 (*generated with MATLAB R2018b,* https://mathworks.com).

**Figure 18:**
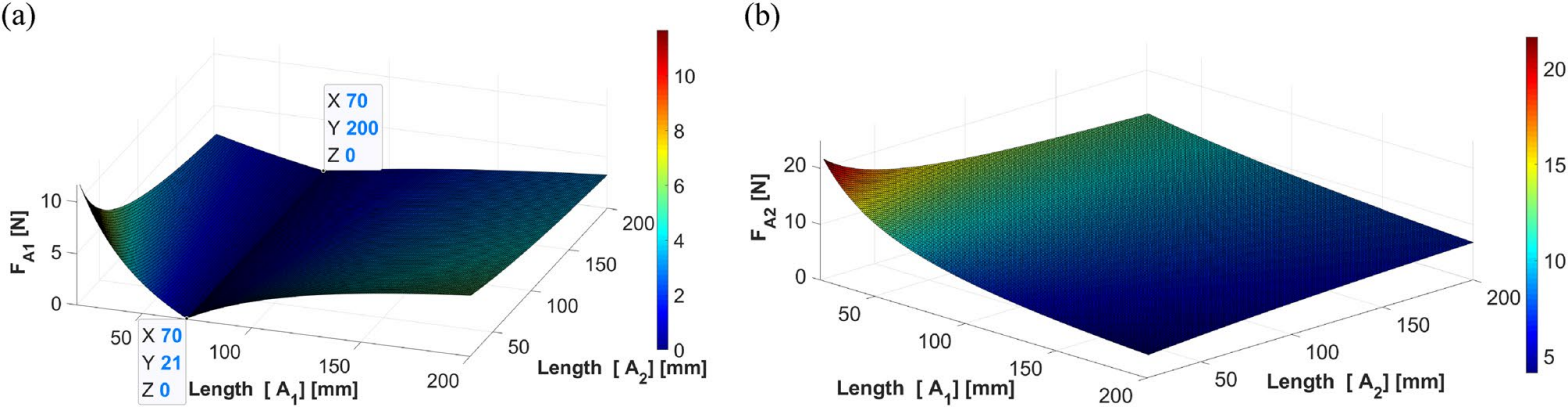
Control force of actuators 1 and 2 when the shaker is placed at 70 mm: (**a**) actuator 1; (**b**) actuator 2 (*generated with MATLAB R2018b,* https://mathworks.com).

**Figure 19:**
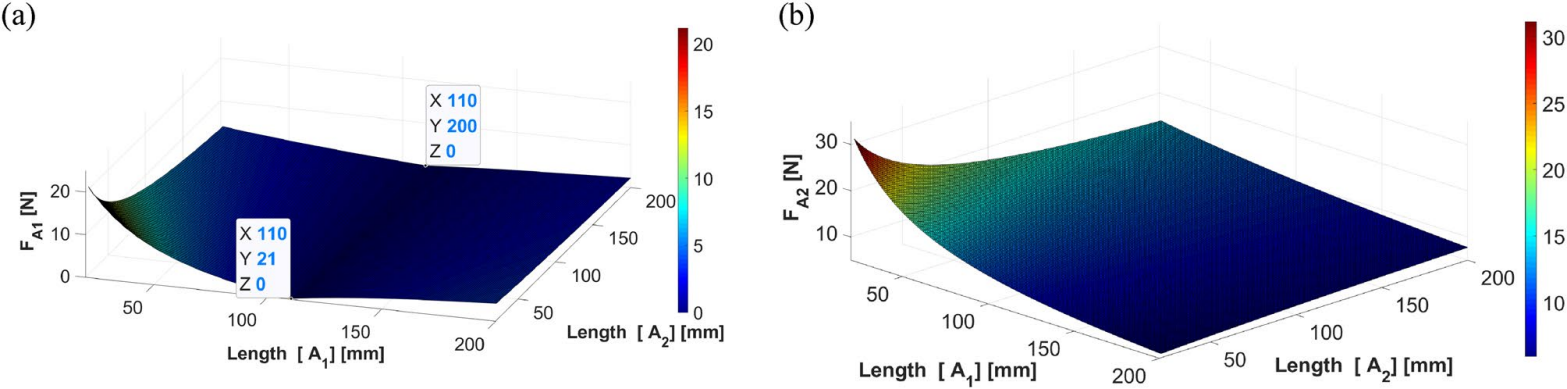
Control force of actuators 1 and 2 when the shaker is placed at 110 mm: (**a**) actuator 1; (**b**) actuator 2 (*generated with MATLAB R2018b,* https://mathworks.com).

Based on the positions of the actuators, the forces in each actuator can be calculated and analyzed using Eq. (26a, b) from Section “Analytical approaches for determining positioning criteria”. Figs. 17, 18 and 19 show the plotting of simulation results when the shaker was located at 40 mm, 70 mm, and 110 mm, respectively. All the results show a trend similar to that of the dynamic simulation results.

Given that the dynamic and static results are similar, it can be assumed that the proposed system can be analyzed by static analysis as follows. ${F}_{A1}$ is obtained using Eq. (26a, b) in Sect.  “Case I: changing the position of actuator 1”. To consider the influence of the changed positions of the actuators, partial derivatives relative to ${A}_{1}$ and ${A}_{2}$ are calculated, as shown in Eq. (27) below.

27 $${F}_{A1}\left({A}_{1},{A}_{2}\right)=\left\{\frac{\left({A}_{2}-S\right)}{({A}_{1}+{A}_{2}{)}^{2}}{F}_{S}\right\}i+\left\{\frac{\left({A}_{1}+S\right)}{({A}_{1}+{A}_{2}{)}^{2}}{F}_{S}\right\}j,$$

To minimize these forces, the following condition, ${A}_{1}=-S$, should be satisfied, such that actuator 1 is in a symmetrical position to the shaker. The other condition, ${A}_{2}=S$, indicates that the locations of actuator 2 and the shaker remain the same.

## Experimental validation

### Experimental setup

**Figure 20:**
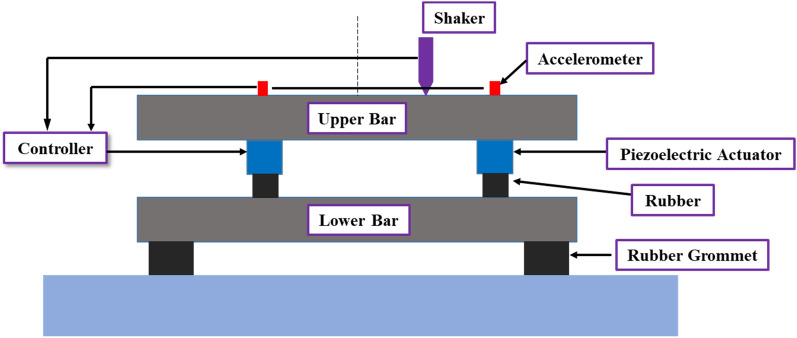
Experimental schematic.

An experiment was conducted to validate the simulation results. A schematic of the experiment is shown in Fig. 20, the upper bar represent the source part would be the engine of vehicle, lower bar is the receiver part would be the sub-frame of vehicle. the rubber and actuator are combined is the path part. It minimizes the perturbation force of the engine and then transmits it to the sub-frame, thereby reducing the vibration of the entire body.

**Figure 21:**
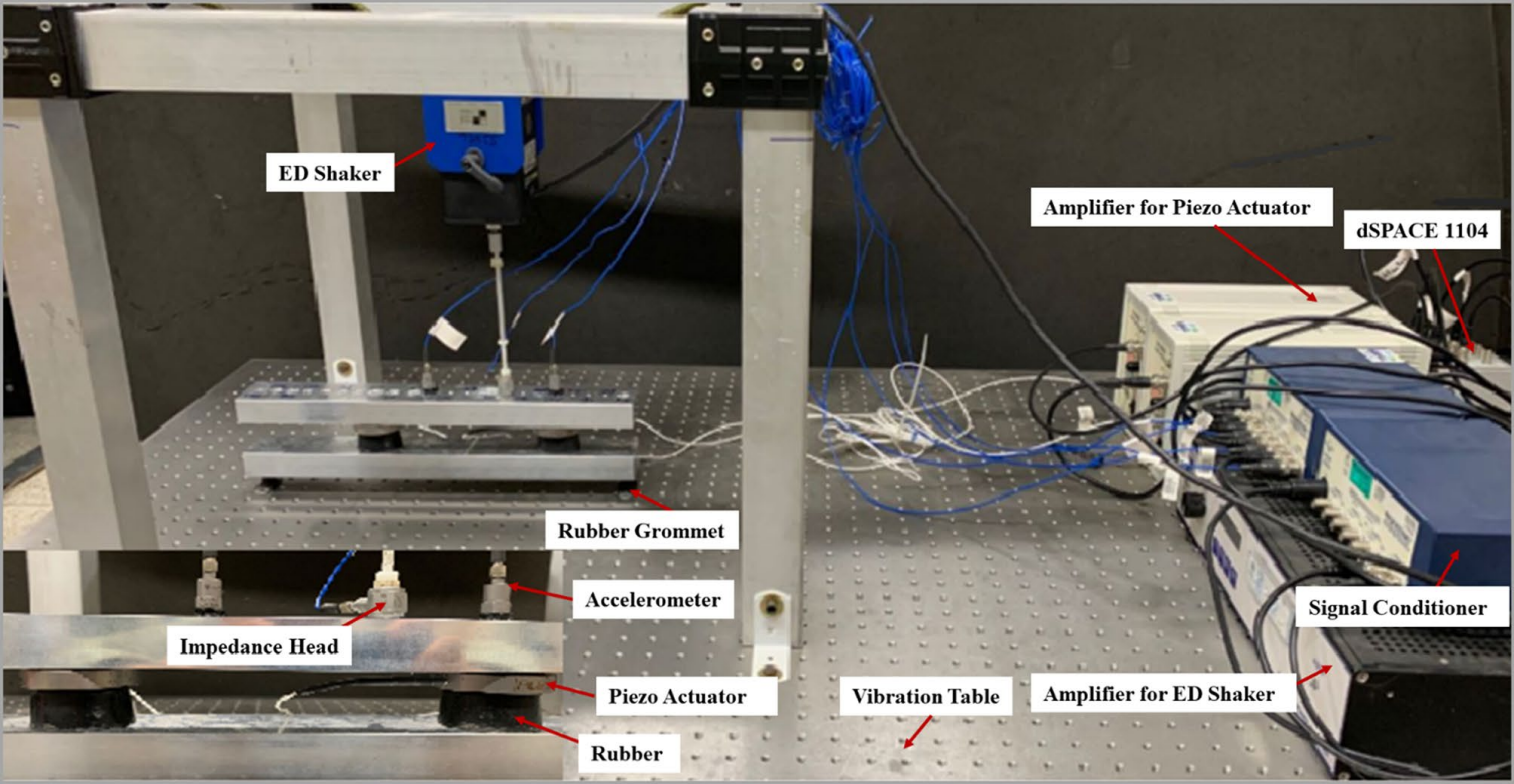
Experimental setup for active vibration control of beam.

The schematic of the experimental setup is shown in Fig. 21. The experimental setup comprised two active paths between the aluminum bars, represent the source part and receiver part. The source and receiver were both 400 mm × 50.8 mm aluminum bars with 25.4 mm thickness, and the active path was connected by a piezoelectric actuator and rubber mounts. The perturbation force from the Electrodynamic shaker, which used a sinusoidal wave at 460 Hz as the input signal. An impedance head attached to the end of the stinger is used to measure the disturbance force. An accelerometer was attached to each actuator, and the signal from the accelerometer was measured in real time using dSPACE. External excitation with a single frequency of 460 Hz was applied using by electrodynamic shaker, and the force was measured using the impedance head attached to the end of the stinger.

The input signal to the actuator was used as the input signal from the aluminum plate accelerometer corresponding to the source, and this signal was operated using an adaptive filter for tracking and then adjusting it accordingly. The experimental results were compared with the simulation results from Section “Two-way variation of active path locations”, and the comparative analysis is described in Section “Experiment results and discussion”.

### Experiment results and discussion

**Figure 22:**
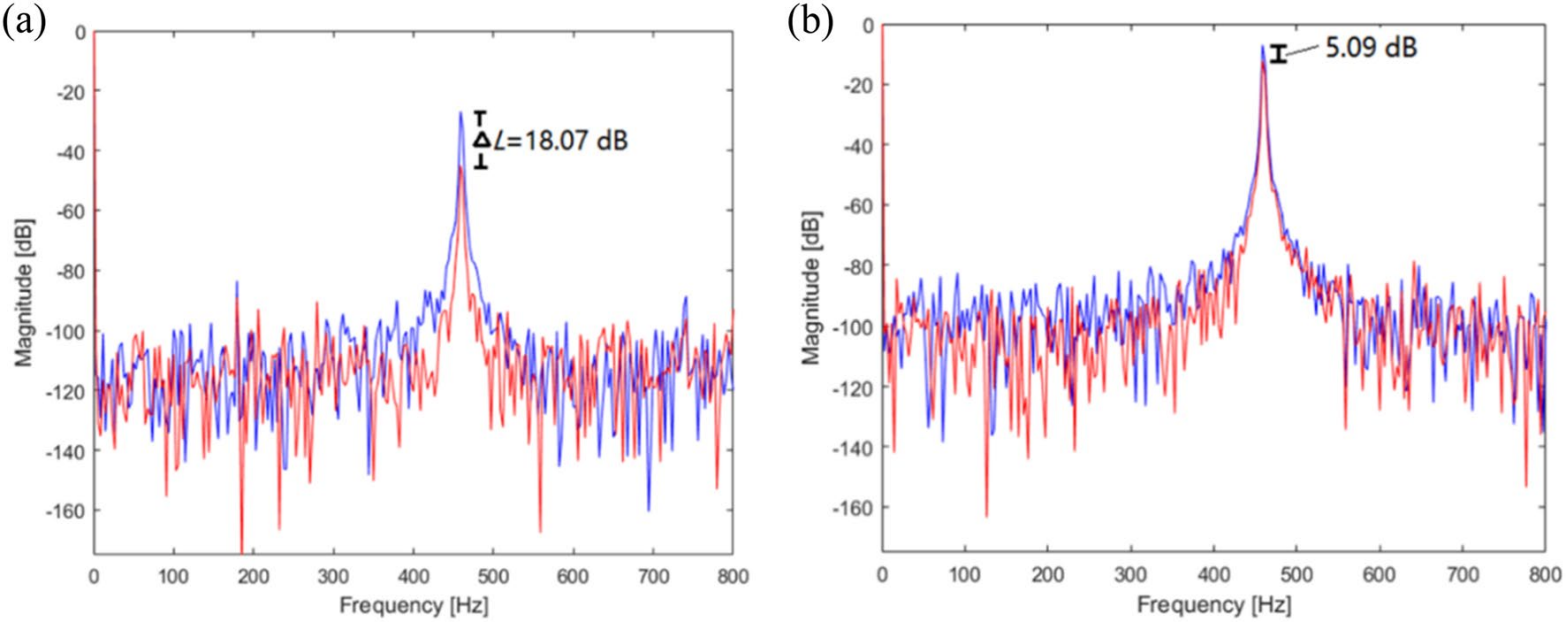
Comparison of measured accelerometer spectra for amplitude sinusoid signals: (**a**) actuator 1; (**b**) actuator 2. Key: blue line uncontrolled; red line controlled.

To validate the simulation results, an experiment comprising six cases was performed, where the position of path 1 was changed. The results from case 2 are shown in Fig. 22 and represent the values when path 1 = 67 mm.

**Figure 23:**
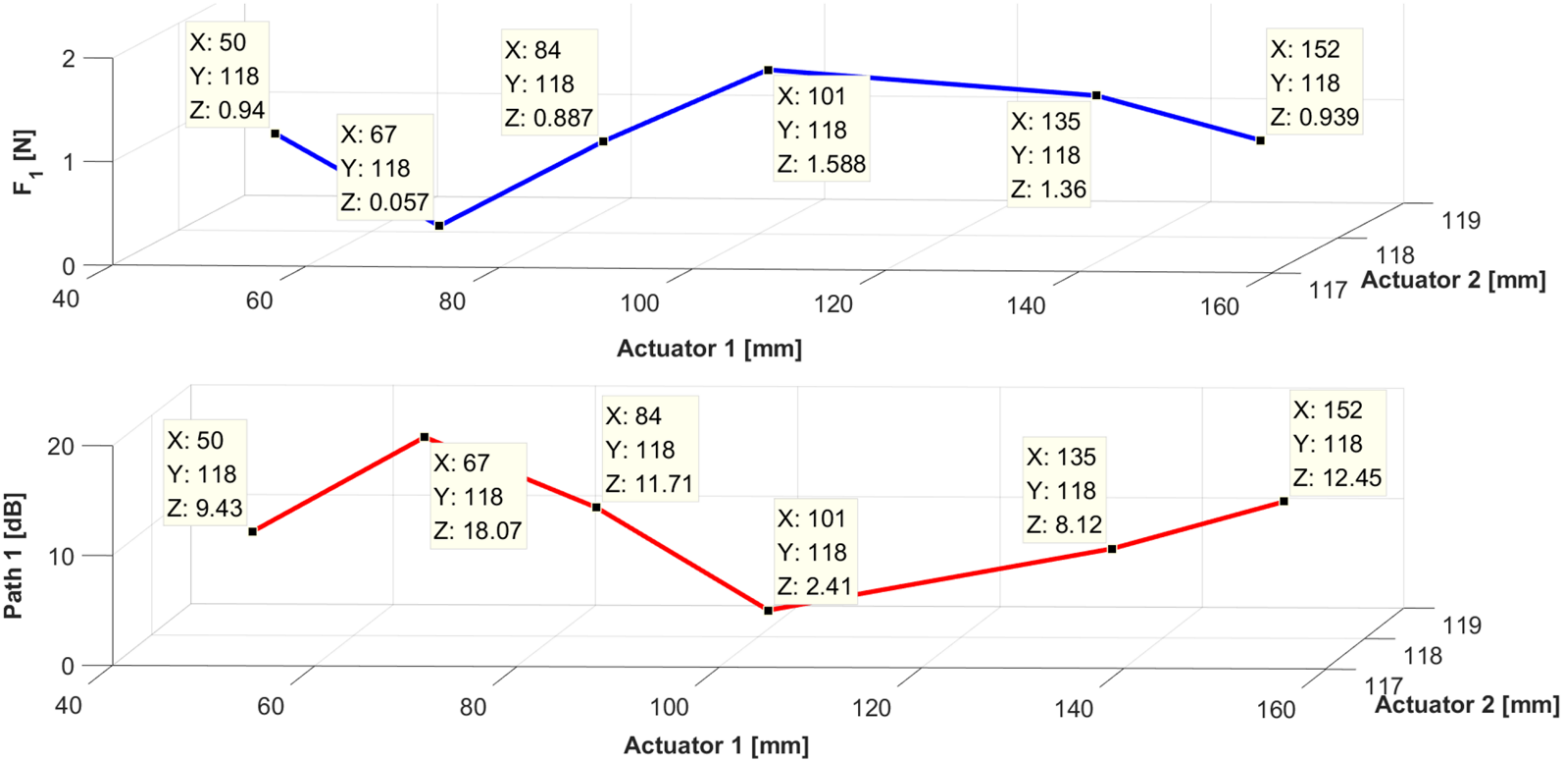
Compared magnitude with displacement for sinusoids at path 1. Key: blue line = control force; red line = vibration reduction.

Fig. 22 shows a graph comparing the frequency spectra of the uncontrolled and controlled spectra. Paths 1 and 2 decreased at 460 Hz to 18.07 dB and 5.09 dB, respectively. Compared with the case where path 1 was placed at 50 mm, the displacement reduction of path 1 was augmented. Moreover, the displacement reduction of path 2 was augmented, as path 1 approached the end of the beam. The results from case 3 are shown in Fig. 23, and it represents the values when path 1 = 84 mm.

**Figure 24:**
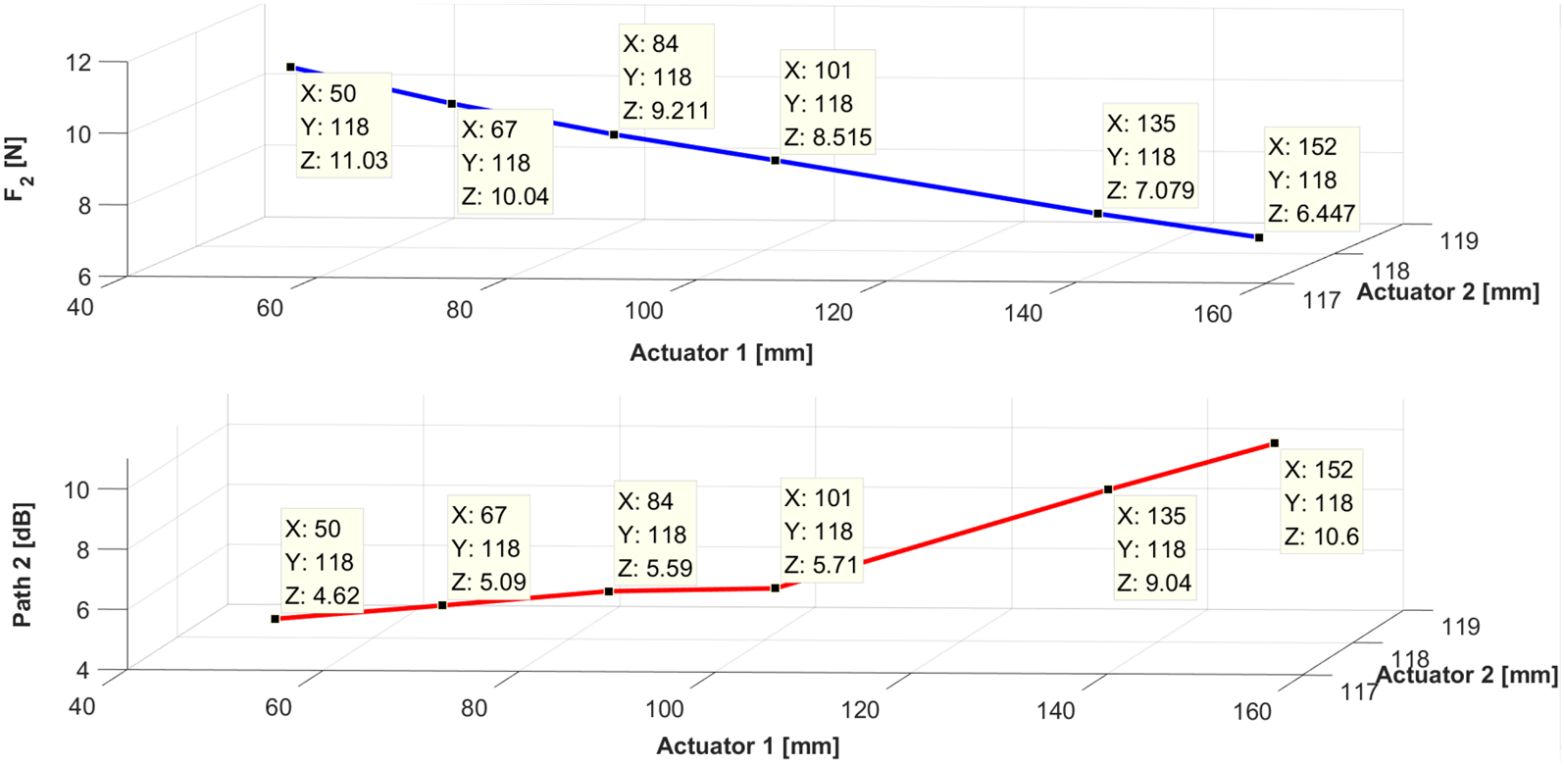
Compared magnitude with displacement for sinusoid signals at path 2. Key: blue line = control force; red line = vibration reduction effect.

Furthermore, the required force and vibration reduction effect corresponding to the change in the location of path 1 are depicted in Figs. 23 and 24. On the basis of Table 3, Figs. 23 and 24, the experimental results can be summarized as follows. (1) The effect of vibration reduction shows notable performance when the required control force is small. (2) Path 2 requires a small control force when path 1 is located near the end of the beam. (3) Path 1 requires only a small control force and exhibits excellent control effect when located at 67 mm. This implies that when path 1 is placed symmetrically at the excitation point, the control effect yields favorable results. In accordance with the summarized results of the simulation and experiment, the optimal placement criteria of the active mounting system on the beam structure are confirmed.

**Table 3:** Reduction of vibration of paths 1 and 2.

Location of path 1	Path 1(peak)	Path 2(peak)	Location of shaker	Location of path 2
50 mm	9.43↓	4.62↓	67 mm	118 mm
67 mm	18.07↓	5.09↓		
84 mm	11.71↓	5.59↓		
101 mm	2.41↓	5.71↓		
135 mm	8.12↓	9.04↓		
152 mm	12.45↓	10.60↓		

## Conclusion

In this study, static and dynamic analyses were performed to determine the optimal position of the active mounting system on a beam structure. Furthermore, an experiment was conducted to validate the simulation results for the optimal position of the active mounting system. The main contributions of this study are as follows: (1) it established the equation of motion for a beam structure, replicating the active path between the vehicle engine and the sub-frame, (2) defined the analytical calculation of the actuator force and phase, (3) suggested the optimal criteria for the active mounting system through simulation, and (4) designed a feasibility experiment setup and validated the suggested criteria.

This study was conducted to determine the active mount position criteria. To identify these criteria, static and dynamic analyses were performed. Consequently, based on the lumped parameter model, an overall beam structure with two active paths was modeled. Furthermore, an analytical calculation method to calculate the secondary force and phase corresponding to the active paths was proposed. The active mounting systems were shifted to equal intervals, and static and dynamic analyses were performed to determine the optimal placement criteria. In the static analysis, when path 1 was located at a symmetrical position to the excitation point, it was confirmed that the smallest control force was required. Furthermore, the required force of path 1 diminished as path 1 moved to the end of the beam. The results were confirmed through dynamic analysis and found to be identical to the results from the static analysis, and a feasibility experiment was performed to validate the results from the simulation. An experiment comprising six different specifications was conducted by changing the location of path 1, and the results demonstrated that the criteria for the optimal position can be suggested through the proposed process. In the future, a plate structure will be employed to optimize locations of vibration paths by considering the motion of both x and y axes.

## Data Availability

The datasets used and/or analysed during the current study available from the corresponding author on reasonable request.
